# *Trichuris muris* as a tool for holistic discovery research: from translational research to environmental bio-tagging

**DOI:** 10.1017/S003118202100069X

**Published:** 2021-12

**Authors:** Iris Mair, Kathryn J. Else, Ruth Forman

**Affiliations:** Faculty of Biology, Medicine and Health, Lydia Becker Institute of Immunology and Inflammation, Manchester Academic Health Science Centre, The University of Manchester, Oxford Road, Manchester M13 9PT, UK

**Keywords:** Ecoimmunology, host–parasite interaction, immunology, population ecology, translational research, *Trichuris*

## Abstract

*Trichuris* spp. (whipworms) are intestinal nematode parasites which cause chronic infections associated with significant morbidities. *Trichuris muris* in a mouse is the most well studied of the whipworms and research on this species has been approached from a number of different disciplines. Research on *T. muris* in a laboratory mouse has provided vital insights into the host–parasite interaction through analyses of the immune responses to infection, identifying factors underpinning host susceptibility and resistance. Laboratory studies have also informed strategies for disease control through anthelmintics and vaccine research. On the contrary, research on naturally occurring infections with *Trichuris* spp. allows the analysis of the host–parasite co-evolutionary relationships and parasite genetic diversity. Furthermore, ecological studies utilizing *Trichuris* have aided our knowledge of the intricate relationships amongst parasite, host and environment. More recently, studies in wild and semi-wild settings have combined the strengths of the model organism of the house mouse with the complexities of context-dependent physiological responses to infection. This review celebrates the extraordinarily broad range of beneficiaries of whipworm research, from immunologists and parasitologists, through epidemiologists, ecologists and evolutionary biologists to the veterinary and medical communities.

## Graphical abstract

**Figure:**
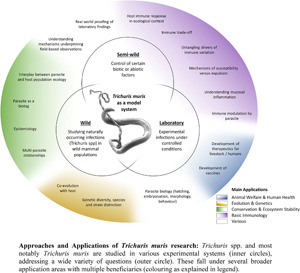

## Introduction

*Trichuris* spp. nematodes are intestinal parasites within the family Trichuridae. There are over 80 known species, infecting a broad range of mammals including wildlife, livestock and humans, causing chronic infections and associated morbidity. The common name for *Trichuris* spp., the ‘whipworm’, has its origins in the characteristic whip-like form of all *Trichuris* species. The lifecycle is similar across these species: embryonated eggs are ingested, hatch in the large intestine and undergo several moults prior to becoming adults. Female adults each shed thousands of unembryonated eggs per day, which are released into the environment within the feces of the host. The eggs then embryonate in the soil where they can remain for years as an infection reservoir. Of all the *Trichuris* species, *Trichuris muris* in a laboratory mouse is the most studied host–parasite system. In this review, we outline the various reasons why *T. muris* has become such a popular model system for a wide range of applications including translational, immunological, genetic and environmental research. We also highlight the gaps in our knowledge and possibilities for future directions using this versatile model organism. Focusing on important discoveries made using the laboratory mouse model we extend these to include insights from studies working with whipworm in wild mammalian hosts. Although laboratory systems have contributed significantly to our understanding of the immunological aspects of host–parasite relationships, ecological studies using wild mammalian hosts have focused on population ecology as well as genetic and evolutionary relationships. Significantly, immunology and ecology are now combining to provide a platform for studying immunology in the wild, providing much needed, context-dependent model systems.

## Understanding immune responses to *T. muris* in laboratory mice: implications for basic and applied science

### Immunity to murine Trichuriasis – a protective Th2-type immune response

Research on *T. muris* has, of course been driven, in part, through a desire to understand more about the parasite and host–parasite interface for translational purposes, but is also used as a biomedical tool to study mucosal inflammation, Th1/Th2-mediated immune responses and immunomodulatory molecules secreted by the nematode. There has also been a wealth of research exploring infection of pigs with *Trichuris suis*. The drivers here are primarily 2-fold: an economic driver, given infection of pigs with this parasite has significant veterinary implications both in terms of animal welfare, meat quality and financial concerns, and a human health angle revolving around a desire to exploit the immunomodulatory properties of *T. suis* to treat human diseases.

There have been several comprehensive reviews published detailing the immunological advances which have been made with *T. muris* (Klementowicz *et al*., 2012; Hurst and Else, 2013; Colombo and Grencis, 2020), therefore we will highlight some of the key findings and novel techniques used in recent years. Historically, it has been well established that Th2 immune responses, characterized by the production of interleukin (IL)-4, IL-5, IL-9 and IL-13 and accompanied by the generation of a parasite-specific immunoglobulin (Ig)G1 response, are vital for the expulsion of the parasite. In contrast, susceptible mice mount an inappropriate Th1-polarized immune response, characterized by high levels of interferon-*γ* and IL-12 and the generation of a parasite-specific IgG2 response which results in persistent infection (Else *et al*., 1994; Artis, 2006; Hadidi *et al*., 2012). Dendritic cells (DCs) have been shown to play a key role in antigen presentation during *T. muris* infections and in recent years the IRF4+ CD11c+ CD11b+ DCs have been identified as the key population for driving a Th2 immune response, whereas in contrast, IRF8+ CD103+ DCs are associated with a Th1 immune response and thus a chronic infection (Luda *et al*., 2016; Mayer *et al*., 2017). The mechanisms by which Th2 effector responses drive the expulsion of the parasite is known, in part, with goblet cells and mucin production identified as key players (Hasnain *et al*., 2010, 2011). In addition, an IL-13 driven increase in epithelial cell turnover is known to drive the parasite from its intracellular epithelial cell niche (Cliffe *et al*., 2005).

During chronic infections, regulation of the intestinal inflammation caused by large burrowing parasites is vital for the prevention of sepsis due to the loss of gut barrier integrity. In *T. muris* infection the regulatory cytokine IL-10 has been shown to be vital in maintaining host survival (Schopf *et al*., 2002). Indeed, mice infected with the Sobreda (S) isolate of *T. muri*s drive an elevated number of regulatory T cells within the intestine. Importantly, a reduction in regulatory T cells during infection through treatment with anti-CD25 antibodies enabled worm expulsion but also demonstrated that these regulatory T cells were vital in protecting the host from intestinal pathology (D'Elia *et al*., 2009).

### The utility of transgenic mouse models in dissecting the immune response against *T. muris*

The use of novel transgenic mouse models has greatly enhanced our understanding of the immunological response to *T. muris* infection. These mouse models included animals where immune cells had been altered, cytokines or cytokine receptors knocked out, mice where cytokines or cytokine receptors were restricted to or absent from certain cell types, or mice where potential effector proteins (e.g. mucins) had been altered. For example, experiments utilizing the IgMi transgenic mouse model, which has B cells that are unable to class switch from IgM and are unable to secrete antibodies, has demonstrated that the B cell plays both antibody-dependent and antibody-independent roles during *T. muris* infection (Sahputra *et al*., 2020). Other approaches have been to target specific cytokines. For example, a recent study has elucidated the role of IL-17A in the promotion of Th2 immune responses in the lung following infection with *T. muris* utilizing global IL-17a knockout mice (Ajendra *et al*., 2020). In addition, the utilization of bone marrow chimeras and the Cre loxP system has further refined these investigations. The Cre-loxP system employs site-specific recombinase technology which allows the removal of genes in specific cell types. Depending on the promoter chosen to drive Cre recombinase, genes of interest can be removed from specific cells for a short time period or permanently. However, experiments involving some Cre drivers have, in part, been haunted by issues of incomplete deletion which are particularly evident under inflammatory conditions and have been well documented for the LysM Cre promoter, originally developed to delete specific genes in macrophages and neutrophils (Vannella *et al*., 2014). Nevertheless, through the use of the epithelial cell-specific Villin-Cre driver, the importance of IL-10 signalling in haematopoietic cells, but not in intestinal epithelial cells, for successful worm expulsion has been established (Duque-Correa *et al*., 2019). Similar approaches have also been utilized to demonstrate the redundant role of Cav1 during infection (Caratti *et al*., 2019) utilizing global Cav1 mutant mice as well as the requirement for Notch expression on basophils (Webb *et al*., 2019) and CXCR5 expression on CD11c+ cells (Bradford *et al*., 2018) in *T. muris* expulsion. The use of transgenic mice has also revealed that the circadian machinery in DCs regulates the Th1/Th2 balance and thus worm expulsion kinetics (Hopwood *et al*., 2018). Wild-type C57BL/6 mice infected late in the day exhibited delayed expulsion kinetics compared to mice infected early in the day, concomitant with a stronger Th2 response in the latter. This time-of-day dependency of both the immune response and expulsion kinetics was abrogated in mice with a DC-specific deletion of the circadian regulator BMAL1.

### The wide variety of interdisciplinary technologies advancing *T. muris* research

In addition to transgenic mouse models, the treatment of mice with depleting antibodies has added to our understanding of the immunological response to *Trichuris*. Studies treating mice with an anti-CD20 monoclonal antibody to deplete B cells have revealed a role for B cells in supporting Th2 immune responses and thus expulsion of *T. muris*, through a yet unknown mechanism (Sahputra *et al*., 2019). In addition to utilizing these transgenic mouse models and depleting antibodies, the depth of understanding acquired from these mouse experiments has been aided through the use of large flow cytometry panels and RNA-sequencing analysis of mutant cell populations (Webb *et al*., 2019; Sahputra *et al*., 2020).

In recent years, significant research has been undertaken into the excretory/secretory products (ES) of *T. muris*. In particular, it has been shown that the whipworm secretes exosome-like extracellular vesicles (EVs) and that these vesicles interact with host cells (Eichenberger *et al*., 2018), are immunogenic (Shears *et al*., 2018) and contain small RNAs (White *et al*., 2020). These experiments have utilized a vast array of different technologies including differential centrifugation for EV isolation followed by turnable resistive pulse sensing analysis, nanoparticle tracking analysis, transmission electron microscopy, dynamic light scattering and proteomics to analyse the EVs themselves (Eichenberger *et al*., 2018; Shears *et al*., 2018; White *et al*., 2020). In addition, a range of technologies have been employed to assess host–EV interactions including RNA and miRNA sequencing, as well as laser scanning confocal imaging (Eichenberger *et al*., 2018). The application of multiple technologies from different research disciplines has also furthered our understanding of specific whipworm molecules. For example, our understanding of the functional significance of p43, long recognized to be the single most abundant protein in *T. muris* ES has benefitted from interdisciplinary research. Recent research studies incorporating confocal microscopy, *in-situ* fluorescent hybridization, electron microscopy, crystallogenesis and X-ray technologies have revealed the immunomodulatory function of the p43 protein (Bancroft *et al*., 2019). Advances in understanding the composition and function of ES is covered in detail elsewhere in this special collection by Bancroft and Grencis (2021).

Whatever the motivator of the research into *T. muris*, we have a clear understanding that a CD4+ Th2 immune response is vital for expulsion of the parasite. However, the exact contributions different cell types provide, and how changes in context may alter these contributions is still to be determined. A deeper understanding of the key drivers of the protective Th2 response is vital in informing the development of potential therapeutics for the parasite. This in itself is complicated by redundancies within the immune system as it is increasingly recognized that the relevance of a particular cell type in infection outcome is largely dependent upon the context. Thus, cellular contributions which may be essential in one mouse strain may be largely redundant in other strains or when the system is artificially modulated. Indeed, how context-dependent protective immune responses translate to humans or other animal species is an area which requires more research.

## The human disease Trichuriasis as a driver for *T. muris* research

*Trichuris trichiura*, the human whipworm, is prevalent in tropical areas with poor sanitation. In areas where it is endemic, infection contributes to chronic nutritional morbidity and poor cognitive development. Moreover, heavy infections can result in complications which include Trichuris dysentery syndrome and rectal prolapse. For a more comprehensive review of human Trichuriasis including its epidemiology, disease mechanisms, diagnosis, screening and prevention, refer to the recently published review by Else *et al*. (2020). *Trichuris muris* is the mouse homologue of the human parasite *T. trichiura*, the aetiological agent of Trichuriasis. Importantly, *T. muris* is a powerful model for *T. trichiura*, as it is a natural parasite of wild mice, demonstrates similar morphology to *T. trichiura*, occupies the same niche within the intestine and displays antigenic cross-reactivity with the human species. Furthermore, the genome is highly conserved between *T. muris* and *T. trichiura* (Roach *et al*., 1988; Foth *et al*., 2014).

### Immunity to human Trichuriasis – a protective Th2-type immune response

Studies on human immune responses to *T. trichiura* are less abundant than those performed using animal models, reflecting in part the challenges that come with studying human immunology in the field. These challenges, which include the presence of multiple coinfections, as well as nutritional, behavioural and genetic heterogeneity between individuals, represent the real-life context within which a host is exposed to *T. trichiura* infections. However, the uncontrolled conditions and multiple variables combine to make data interpretation difficult (Else *et al*., 2020). Nevertheless, fieldwork has provided clear evidence for strong correlations between increased Th2 immune responses and reduced infection intensities, with these Th2 protective immune responses slowly developing over years of exposure. For example, the levels of *T. trichiura*-specific IgE increase as worm burdens decrease with age (Faulkner *et al*., 2002). Such serological data are substantiated by analyses looking at the balance of CD4+ T helper cell cytokine responses in the context of soil-transmitted helminths in general (Turner *et al*., 2003; de Ruiter *et al*., 2020) which also place the Th2 immune response as central in the control of infection. Thus, in the study by Turner *et al*. (2003), Th2 cytokine levels in the periphery increased as worm intensities decreased. These data were however only significant in the older age cohorts of subjects, suggesting that acquired immunity to infection develops after years, possibly decades, of exposure to the parasite. Beyond field work, self-infection studies, which are limited in sample size, have also pointed towards *T. trichiura* infections promoting Th2 immune responses over time (Broadhurst *et al*., 2010; Dige *et al*., 2017). For example, Dige *et al*. (2017) used a single individual infection study to explore the local mucosal and systemic immune responses post *T. trichiura* infection. Colonic mucosal biopsy profiles evidenced mixed T helper cell responses across Th1, Th2, Th17 and T regulatory cells, whereas the systemic immune response clearly shifted towards Th2 dominance over time.

### Current treatments, the need for novel solutions and the role of *T. muris* in their development

Current treatment of human Trichuriasis relies on preventative chemotherapy, with water and sanitation programmes as well as education as important adjuncts (Else *et al*., 2020). However, anthelmintic treatment options for Trichuriasis are poorly efficacious and the drug development pipeline is sparse. The WHO currently recommends the use of albendazole, mebendazole, levamisole and pyrantel pamoate for the treatment of soil-transmitted helminths. However, the cure rates for these drugs are unacceptably low against *T. trichiura* with mebendazole showing the highest efficacy (44%) followed by albendazole (32%), levamisole (29%) and pyrantel pamoate (23%) (Moser *et al*., 2019). Importantly, modelling studies demonstrate that these poor cure rates are insufficient to interrupt *Trichuris* transmission and are also too low to eliminate morbidity from *Trichuris* infection (Turner *et al*., 2016). In order to overcome these limitations there has been an increasing interest in the development of drug combination therapies. The combination of albendazole and ivermectin, which are both approved for use in humans, demonstrated a cure rate of approximately 60% (albeit with limited data collected on the efficacy) (Palmeirim *et al*., 2018), however, more data are required on the safety of this combination and unfortunately it is contraindicated in areas where the filarial parasite *Loa loa* is prevalent, thus limiting the scope of this solution.

All the current and proposed drug combinations used by mass drug administration programmes rely on benzimidazole drugs (albendazole or mebendazole). A number of small studies have demonstrated the presence of benzimidazole-resistant tubulin polymorphisms in *T. trichiura* with an increased frequency evident following treatment, and these polymorphisms are associated with poor benzimidazole efficacy (Diawara *et al*., 2013). Thus, there is an urgent need for both widespread monitoring of these benzimidazole-resistant alleles but also novel compounds which target the parasite through alternative mechanisms. *T. muris* in the mouse has featured significantly in basic research motivated by the need for novel drugs. Encouragingly, in recent years novel compound series have been proposed as anthelmintics including 2,4-diaminothieno[3,2-*d*]pyrimidines and dihydrobenzoxazepinones, both of which show efficacy against not only the adult parasite, but also the egg stages and thereby may enable a break in the parasite lifecycle (Partridge *et al*., 2017, 2018). In addition, and again using *T. muris* in the mouse, plant-based compounds have been investigated as new anthelmintics (Stepek *et al*., 2006; Wangchuk *et al*., 2016).

Vaccination is one of the greatest advances in global health; however, limited progress has been made in vaccine research focused on human Trichuriasis despite the existence of the powerful mouse model. Given the wealth of knowledge on the immunological response to the parasite, the limited progress made in vaccine generation, for example in comparison with hookworm, is somewhat surprising. When looking back over the last 20 years, a PubMed search of the words ‘Trichuris’ and ‘vaccine’ yields only 59 results in comparison with 311 when searching for ‘hookworm’ and ‘vaccine’. The reason for this discrepancy is not clear, and cannot be accounted for by when the genomes of the parasites were available as these were all published in 2014–2015 (Foth *et al*., 2014; Jex *et al*., 2014; Tang *et al*., 2014; Schwarz *et al*., 2015). One possible explanation may be the level of morbidity driven by the different parasites – the disability-adjusted life-years for Trichuriasis (213 000) is estimated to be approximately one-quarter of the number observed in hookworm (845 000) (Kyu *et al*., 2018). Nevertheless, the need for a vaccine is clear, especially given the climate of emerging infectious diseases and the impact of STH infections on susceptibility to other pathogens and co-morbidities (Salgame *et al*., 2013) (see also Hayes and Grencis, 2021 within this special issue). Vaccine development is discussed in detail by Hayon *et al.* (2021) within this special issue.

## Understanding parasite biology – *Trichuris* research of basic, applied and ecological importance

Despite the widespread use of especially *T. muris* in biomedical research, there is a lack of understanding of the parasite itself. *Trichuris* is a soil-transmitted enteric helminth, and although specific life cycles are likely slightly variable, the general lifecycle of *Trichuris* spp. is well known (Hurst and Else, 2013). Embryonated eggs are ingested through contaminated food or water, and eggs hatch in the large intestine. In the caecum and proximal colon, L1 larvae burrow into epithelial cells of the gut and moult to the second larval L2 stage. Both L1 and L2 larvae live intracellularly. From the L3 stage onwards, the posterior end protrudes into the gut lumen, and the thinner anterior end, containing the stichosome, burrows tunnels through epithelial cells. Unembryonated eggs, between 2000 and 8000 per day from adult females, are shed with the feces. These eggs then embryonate in the soil, depending on environmental conditions in roughly 2–4 weeks.

### How do eggs embryonate?

**Fig. 1.**
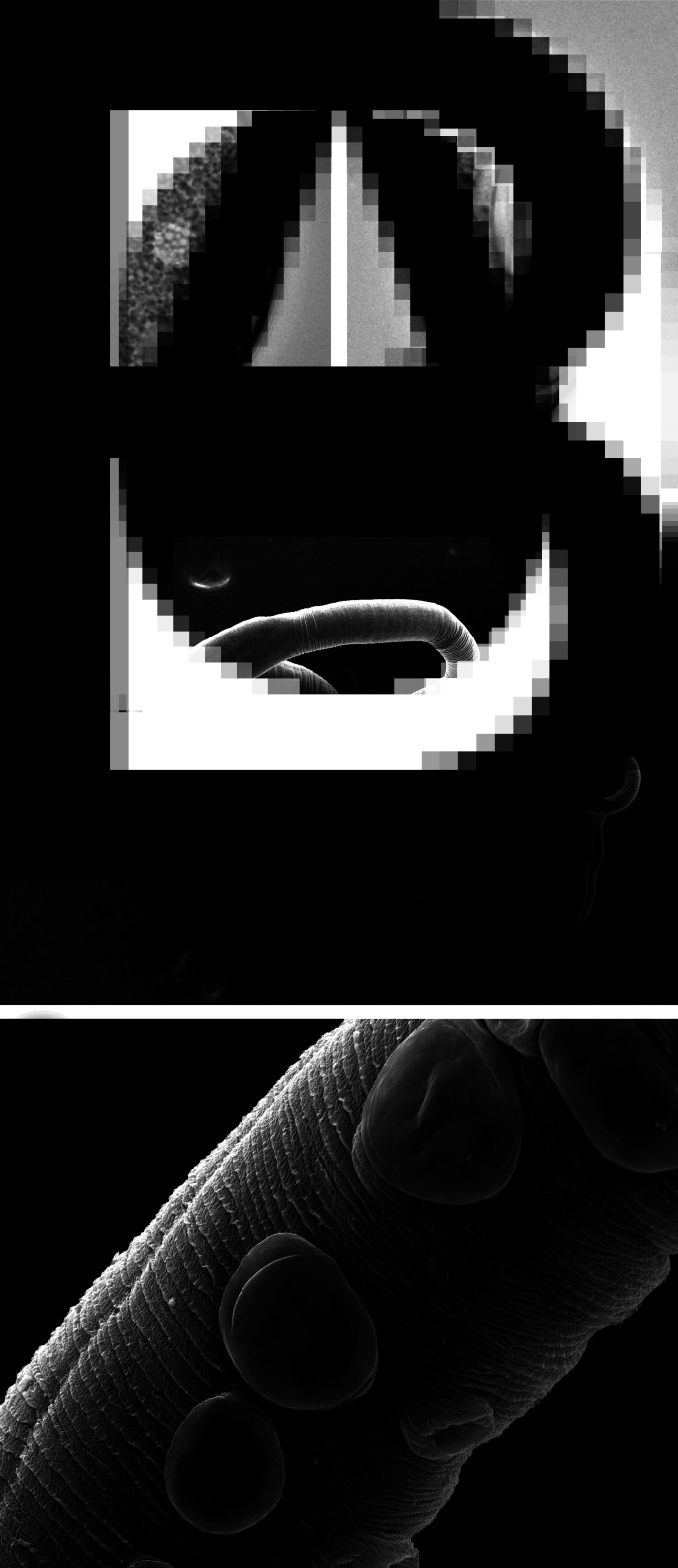
*Trichuris muris* lifecycle stages. *Trichuris muris* unembryonated (a) and embryonated eggs (b) taken by light microscopy. Scanning electron microscopy images of an adult *T. muris* worm (c), as well as the cuticular inflations visible along the bacillary band (d).

Similarly to the sparsity of knowledge on the moulting process *in vivo*, there is also a lack of data on the *Trichuris* egg embryonation process (Fig. 1a and b). Indeed, the majority of the literature on *Trichuris* spp. egg embryonation was reported in the early 1900s. These studies, which have primarily been performed in aqueous media, have demonstrated that eggs take up to 60 days to embryonate when incubated at 21°C and subsequently remain infective for at least 5 years (summarized in Brown, 1927). Brown additionally studied egg development in soil, which revealed impacts of humidity, temperature and soil type with *T. trichiura* eggs embryonating in 21 days when utilizing optimal conditions. In addition, these studies demonstrated that sandy soils and high temperatures were unfavourable for egg development. Subsequent research on *T. muris* records that the first outlines of the larvae appear after 22–23 days when eggs are maintained at 25–26°C and the fully grown larvae are present following 30–31 days of incubation (Fahmy, 1954). Interestingly, since the study of Fahmy (1954), our understanding of egg embryonation has been primarily furthered *via* studies using the pig whipworm *T. suis* rather than *T. muris*, including detailed morphological observations of the stages of embryonation (Beer, 1973). In recent years, the embryonation of *T. suis* has been investigated in more detail due to the therapeutic potential of *T. suis* ova (or TSO) (Huang *et al*., 2018; Fleming *et al*., 2019; Hollander *et al*., 2020). Data from these experiments have demonstrated that an increase in temperature will drive an accelerated embryonation process; however, at temperatures above 40°C they become degenerate (Vejzagić *et al*., 2016). Embryonation of species other than *T. suis* has not been described in detail and moreover, an understanding of the impact of external factors such as soil ecology on this process is an area of clear need, since preventing the embryonation process could interrupt the lifecycle of the parasite.

### Egg-hatching signals from the microbiome – the lessons learnt from the laboratory mouse model

The hatching process of *Trichuris* is incompletely understood, but most knowledge has been gained through studies on *T. muris*. Hatching of the most commonly used laboratory strain of *T. muris*, the Edinburgh (E) isolate, is dependent on the presence of certain microbiota species (Hayes *et al*., 2010), whereas bacteria-independent hatching has been proposed for the S isolate (Koyama, 2013). Between species differences have also been observed; *in vitro* hatching conditions with bacteria that are successful for *T. muris* have proven unsuccessful for *T. suis* (Vejzagić *et al*., 2015). Host microbiome composition, in turn, is modulated by the nematode (Broadhurst *et al*., 2012; Li *et al*., 2012; Holm *et al*., 2015), a shift that can be reversed by anthelminthic treatment (Houlden *et al*., 2015). Interestingly, this shift in microbiome composition has been shown to suppress the hatching of further ingested eggs, thus providing a host–immune system independent mechanism by which the established parasites may control parasite numbers (White *et al*., 2018). Interestingly, the parasite itself develops its own specific microbiome which it acquires from the host (White *et al*., 2018). The role of the microbiome in shaping parasite–host interaction in the context of *T. muris* is becoming increasingly evident, and a detailed review on this topic is presented by Lawson *et al*. (2021) in this special issue. It is as yet unclear how much these processes are transferrable across different *Trichuris* spp. and host species.

### Visualizing whipworm morphology and uncovering whipworm behaviour

The general morphology of *Trichuris* is well described, with the whip-like form of all *Trichuris* species bestowing on the worm its common name (Fig. 1c). However, whipworm behaviour *in vivo* is less well studied. One of the unresolved mysteries of this macroparasite is how to marry the low level of inflammation and damage response induced in the host, with the fact that the nematode burrows through epithelial cell layers, thereby forming a tunnel. However, the advancement in imaging techniques is opening new avenues to fill this knowledge gap and again focuses primarily on *T. muris* in the mouse. Between the L2 and L3 stages, *T. muris* develops so-called cuticular inflations (Wright, 1975), anterior-ventral projections of the cuticle along the bacillary band, the function of which is still unknown (Fig. 1d). As these cuticular inflations are on the part of the worm that forms the tunnel, it is hypothesized that they might be required to maintain the intracellular niche in close contact with the host. The nematode cells underlying the inflations have been shown to contain many mitochondria, and are therefore suggested to perhaps aid active transport (O'Sullivan *et al*., 2021). In addition to the enigmatic cuticular inflations, the bacillary band as a whole has also been the focus of research. The bacillary band is located ventrally, on the anterior portion of the nematode and comprises longitudinal rows of bacillary cells which lie underneath bacillary pores. The function of the bacillary band is still under debate. Again using state-of-the-art imaging techniques, as well as metabolic assays, bacillary band cells are hypothesized to be involved in secretory as well as absorptive processes (Hansen *et al*., 2016; Lopes-Torres *et al*., 2020).

Although the majority of adult worms position their anterior end within epithelial cells at the surface of the crypts adjacent to the gut lumen, a recent study in laboratory mice, using 3D X-ray micro-computed tomography, found that a subset of *T. muris* burrow down the crypts towards the stem cells at the crypt base (O'Sullivan *et al*., 2021). The progress in imaging techniques as exemplified above may produce new insights into parasite behaviour enabling us to answer unknown questions including their so far unknown feeding behaviour (O'Sullivan *et al*., 2018).

### Advances in the genetics and understanding of host specificity of *Trichuris* – what can we learn from studying whipworm in the wild?

The review so far has focused on studies using the laboratory mouse and controlled model systems, primarily driven by medical and veterinary research. Study of naturally occurring *Trichuris* infections, on the contrary, allows us to understand more about parasite genetic diversity and the co-evolution of parasite genetics and host genetics.

As mentioned earlier, over 80 *Trichuris* species have so far been identified, and the number is growing every year. Most are considered host species-specific, although more recent molecular analyses are challenging this paradigm, and *Trichuris* may rather be considered specific to a taxonomic group. *Trichuris* species have traditionally been differentiated by morphological classification such as length of spicule sheath or structure and orientation of female sex organs (Chandler, 1945); however, some of the *Trichuris* species cannot be differentiated from one another through morphological characterization (Gagarin, 1974). The development of genetic analyses has therefore paved the way for more accurate delineation of species and sub-species strains. Internal-transcribed spacers (ITSs) of nuclear ribosomal rDNA can be used as genetic markers for the identification of closely related nematode species (Campbell *et al*., 1995; Chilton *et al*., 1995; Hoste *et al*., 1995), and ITS sequences in combination with other genetic markers have helped to clarify the distinction of several *Trichuris* species and their varied hosts (Oliveros *et al*., 2000; Cutillas *et al*., 2002, 2004, 2009; Johnston *et al*., 2005; Liu *et al*., 2012; Callejón *et al*., 2013; Xie *et al*., 2018). As the technology is not universally available or applied, however, even recent descriptions of new species are often based on morphological analyses (Del Rosario Robles *et al*., 2006; Purwaningsih, 2013; Robles and Navone, 2014; Robles *et al*., 2014). In the discussion below, we therefore just refer to ‘*Trichuris*’ when discussing publications in which species attribution cannot be ascertained.

Historically, *T. muris* was thought to be the sole *Trichuris* species infecting both murid (including *Mus musculus* and *Apodemus sylvaticus*) and arvicolid (including *Microtus agrestis* and *Myodes glareolus*) hosts from the superfamily Muroidae, but isoenzymatic analysis (Feliu *et al*., 2000) and later ITS sequencing (Cutillas *et al*., 2002) identified *Trichuris arvicolae* as a distinct *Trichuris* species. However, both groups reported the respective *Trichuris* species to infect several hosts within their taxonomic group. This highlights that spill-over between species may be a common occurrence, adding to conservation and health concerns. Indeed, several, but not all, *T. trichiura* strains can infect both humans and non-human primates, the latter being a potential (untreated) reservoir for this parasite as well as infected humans being a potential source of infection for wildlife (Ghai *et al*., 2014). Several further comparative studies of related host species (Callejón *et al*., 2013; Xie *et al*., 2018; Cavallero *et al*., 2019), or host species in different geographical locations (Hawash *et al*., 2015), have also shown genetic variability as well as differing infection patterns amongst *Trichuris* strains of the same *Trichuris* species, showing that the taxonomy of this parasite is more complex than previously appreciated.

The relatedness of different *Trichuris* strains from the same *Trichuris* species informs us about both parasite evolution and potential co-evolution with the host. Wasimuddin *et al*. utilized a natural divergence in host genotype to study the potential *Trichuris* ‘intimacy’ with its host (Wasimuddin *et al*., 2016). Although in Western Europe, *M. musculus domesticus* is the prevalent house mouse species, in Eastern Europe, it is *M. musculus musculus*. Where these genotypes meet, there is a hybrid zone which creates a strong central barrier to gene flow between the mouse taxa, as the hybrid males are sterile (Payseur *et al*., 2004). However, *T. muris* genomes did not match the host genome geographical pattern (Wasimuddin *et al*., 2016), in contrast to another endoparasite, *Syphacia obvelata* (Goüy de Bellocq *et al*., 2018), the protozoan parasite *Cryptosporidium tyzzeri* (Kváč *et al*., 2013) and the murine cytomegalovirus (Goüy de Bellocq *et al*., 2015), whose genetic structures all reflected that of their host. The lack of host–parasite co-evolution in the case of *T. muris* is thought to be due to the abundance of alternative hosts in the same area (Wasimuddin *et al*., 2016).

Overall, the accumulating evidence of high species diversity and within-species diversity of *Trichuris* spp. makes it more complex to delineate distinct groups – where do strains end and species start? The increased genetic complexity also begs the question whether treatment and mitigation strategies may not universally apply.

## Studying naturally occurring infections with *Trichuris* spp. – a tool for ecological and environmental research

Beyond its implication in human and livestock health, *Trichuris* species also affect wildlife, and form an integral part of ecosystems. There is therefore a considerable amount of research directed towards understanding the relationship between the parasite and host population ecology, whether with a view to conservation, biodiversity or pest control. Simultaneously, *Trichuris* spp. and especially *T. muris* give opportunities as a model system in natural settings to understand biological processes unrelated to the disease itself, such as parasite–parasite interactions and ecosystem stability.

Parasitic nematode infections including *Trichuris* can have a profound impact on host reproductive success, physiology and behaviour (Dobson, 1988; Wren *et al*., 2021). They can thereby be modulators of natural ecosystems and need to be taken into consideration in biodiversity and ecosystem functioning research (Frainer *et al*., 2018). Studying wild systems can also serve as testing grounds for the validity of hypotheses generated in laboratory experiments, whether they hold true in the more complex, uncontrolled environment. Vice-versa, relationships seen in the wild can be tested mechanistically in the laboratory. Although many endo- as well as ectoparasites have been studied in the wild to such purpose, we summarize here some *Trichuris*-related examples.

### Co-infections in wild rodent populations

Wild host populations typically harbour more than one parasite species. Although co-infections and their frequent mutualistic or antagonistic relationships are also being studied in a laboratory context, the interaction between these co-infections and their host can be shaped or masked by other environmental variables (reviewed by Mabbott, 2018). So far, studies have found anything between none (Behnke *et al*., 2005) and multiple (Fenn *et al*., 2020) associations between *T. muris* and other parasite species infecting murine host species, while taking into account relevant factors such as sex, proxy of age, site, year and season. Different infection prevalence overall, distinct environmental influences, as well as choice of appropriate analysis tools are likely to account for these contrasting conclusions. Noteworthily, in the latter study, *Trichuris* and the pinworm *S. obvelata* showed a strong negative correlation, a relationship that has also been proposed by lab studies (Keeling, 1961). This study of a wild population of house mice therefore confirms that even in the presence of many uncontrolled host–extrinsic and host–intrinsic variables, a negative interaction between these two parasites prevails.

### *Trichuris* epidemiology

The general distribution of *Trichuris*, namely an overdispersed distribution, with the majority of individuals harbouring low levels of worms and a few individuals having high worm burdens, appears consistent between wild animal species (Behnke *et al*., 1999; Fenn *et al*., 2020) and humans (Croll and Ghadirian, 1981; Bundy *et al*., 1987). As expected due to the transmission method of *Trichuris*, very young wild mice are consistently reported to have no or low worm burdens. However, whether infection prevalence or worm burden increase with age in general is debated (Behnke *et al*., 1999; Fenn *et al*., 2020). An important factor is that field studies often use a proxy for age, which differs amongst different studies and may therefore lead to inconsistent results.

In humans, worm burdens spike during early life, then decrease to a low level in older age cohorts (Bundy *et al*., 1987; Needham *et al*., 1992; Faulkner *et al*., 2002), probably reflecting an acquired immunity over time (Turner *et al*., 2003; Broadhurst *et al*., 2010; Dige *et al*., 2017). However, due to the relatively short lifespan of wild rodents compared to their potential life span in a controlled environment, one can argue that the wild rodents studied do not get to an ‘old’ age, since most animals die over winter and the parasite could theoretically outlive the host. Nevertheless, the matching population-level distribution pattern of *T. muris* in wild mice makes this an ideal candidate to study host susceptibility and population dynamics.

### Host susceptibility and population dynamics – learning from the laboratory and the wild

High *Trichuris* burden in wild rodents has been linked to females that are lactating or pregnant in several study populations (Behnke and Wakelin, 1973; Sanchez *et al*., 2011). This phenomenon has been confirmed in laboratory studies (Selby and Wakelin, 1975), and is suggested to be due to suppression of the immune response in these animals which does not allow the same control of the parasite as under other reproductive conditions. Beyond factors such as sex, reproductive status or age, a major factor affecting host susceptibility is genetics (Williams-Blangero *et al*., 2002), in part determined by major histocompatibility complex (MHC) alleles (Froeschke and Sommer, 2012). Laboratory studies take advantage of the fact that *T. muris* induces very different immune responses and associated susceptibility patterns in different commercially available inbred mouse strains in order to understand more about beneficial or detrimental immune responses to infection, attributed both to MHC-II alleles and other genes (Else and Wakelin, 1988; Else *et al*., 1990).

The intricate connection between parasite and host population dynamics also allows the use of parasite data to infer knowledge about the host population as well as its associated ecosystem. For example, *Trichuris* infections have the capacity to control population growth in several rodent species and may therefore represent an in-built pest control (Deter *et al*., 2008), or raise conservation concerns, depending on the viewpoint. On the contrary, rodent parasites (including *Trichuris*) have been successfully used as a biological tag to assess the process of ecosystem regeneration in a burned area in the years following the fire, by comparing parasite prevalence and abundance to an adjacent unburned area (Sáez-Durán *et al*., 2018). Due to the dependency of *Trichuris* both on host behaviour and population structure as well as on environmental conditions due to egg embryonation occurring outside of the host, it can serve as a sentinel for disruptions in the host population. Monitoring parasite prevalence or biology (e.g. in the case of heavy metal accumulation) in areas with natural disturbances, often of anthropogenic nature, is a promising way to indirectly assess ecosystem stability or recovery (Marcogliese and Cone, 1997; Sures, 2004; Sures *et al*., 2017).

Although the conclusions drawn from natural systems may be particular to a specific study population due to their unique environment, genetic make-up, physiology and behaviour, these ‘natural laboratories’ offer opportunities to understand real-life processes that are still largely untapped, when compared to the wealth of *Trichuris* research performed and knowledge gained in laboratory-based studies.

## Bridging the gap – semi-controlled studies of *Trichuris*

The integration of knowledge gained through controlled laboratory studies and the data generated from experiments performed in natural settings is often difficult. However, by combining the multi-factorial, observational approach of ecological studies with the mechanistic, interventional approach of laboratory studies, new study systems are emerging that tackle causation while embracing environmental and/or genetic variability. This is leading to new insights as well as validation of connections established through controlled laboratory studies. In particular, wild house mouse populations offer the combined wealth of laboratory analytical tools and knowledge base, as well as the incorporation of ecological parameters. Moreover, with advances in non-species-specific analytical tools, statistical analysis and computational power (Flies, 2020), wild studies of non-model organisms are able to integrate a growing number of variables in their analyses. One great example of an upcoming research area that is pushing the boundaries between the laboratory and the wild is the field of ecoimmunology, and our understanding of parasite–host interaction, especially of *T. muris*, is quickly expanding due to efforts in this field.

### Interventions in wild rodent populations infected with *Trichuris*

For decades, researchers have used interventions in wild study populations in order to ascertain not only correlation, but causation within these complex biological networks. Wild caught wood mice experimentally infected with *T. muris* showed a comparable infectivity pattern as laboratory mice (Behnke and Wakelin, 1973). As is seen in the laboratory mouse, infection with a low dose of *T. muris* eggs resulted in the establishment of a chronic infection where as a high dose resulted in an acute infection which was expelled prior to the worms reaching adulthood. The study concluded that qualitatively similar immune responses must be elicited in both wild and laboratory mice, and wild rodent populations probably acquire the high prevalence of low level *Trichuris* infections through the ingestion of a few eggs.

Using wild-captured voles, another study determined that *Trichuris* infection resulted in reduced pup mass (Deter *et al*., 2007), suggesting a trade-off between the response to infection and investment in the offspring. As mass at birth is a predictor of juvenile survival as well as influencing future breeding (Koskela, 1998; Koivula *et al*., 2003; Ylönen *et al*., 2004), this connection is likely to affect population dynamics.

The reverse approach has also been used, namely the application of anthelmintics in wild animal populations. Using this approach, and selectively treating either females or males, Sanchez *et al.* established that, in a common vole population, females made a greater contribution to the spread of *Trichuris* than males (Sanchez *et al*., 2011). Females in this study had overall higher prevalence of *Trichuris* as well as higher fecal egg counts than males. Instead of an inherent sex bias *via* physiology, the mechanism proposed was a difference in behaviour; females in this population share communal nests and have overlapping territories, feed more during lactation and are the main burrowers, and therefore may be at an increased risk of exposure to this soil-transmitted helminth.

The above studies focus mainly on the impact and dynamics of *Trichuris* infection at a population level, rather than the individual level. In order to investigate the individual's immune response to *Trichuris* in the context of its natural environment, the house mouse in combination with *T. muris* offers itself again as the ideal model system. Laboratory mouse-based studies have been used to identify single intrinsic and extrinsic factors which influence susceptibility. For example the gender and age of a mouse alters the outcome of infection (Humphreys and Grencis, 2002; Hepworth and Grencis, 2009; Hepworth *et al*., 2010) as do diet, time of infection, level of infection and infection regime (Michael and Bundy, 1991; Bancroft *et al*., 1994, 2001; Hopwood *et al*., 2018; Glover *et al*., 2019). Although studying each of these variables in isolation has provided significant insight, wild immunology systems offer opportunities to gain much greater insights in to how multiple variables combine to determine infection outcome.

### Re-wilding laboratory mice in outdoor enclosures

A relatively novel approach to investigating the immune response to *Trichuris* in a semi-natural context is to release laboratory mice into an outdoor enclosure to allow environmental factors to shape the host's response to parasitic infection while controlling the genetic makeup. Using such system, Leung *et al*. demonstrated that the well-known resistance to *T. muris* in laboratory C57BL/6 mice when given a high dose of infection is abrogated when these mice are kept under more natural conditions (Leung *et al*., 2018). This was associated with enhanced microbial diversity and increased Th1 and decreased Th2 immune responses in the gut lamina propria and mesenteric lymph nodes. This means that a ‘resistant’ genetic make-up in the laboratory was turned into a ‘susceptible’ one through environmental influences. This type of semi-controlled study system has the potential to dissect out the contribution of genetics *versus* environment and which environmental factors are the key drivers for observed immune phenotypes and health outcomes.

Using a similar experimental layout, a later study showed that the diversification of the microbiome that occurred following the exposure to a natural environment was altered by infection with *Trichuris*. Changes in several bacterial families associated with outdoor transition were buffered by the helminths, whereas the increase of two taxa acquired in the outdoor enclosure was amplified (Bar *et al*., 2020). The involvement of the microbiome in the maintenance of health is becoming increasingly apparent, therefore, being able to investigate how certain host–extrinsic factors impact microbiome diversity in a semi-controlled setting will be a valuable addition to laboratory and human research. Releasing laboratory mice into semi-natural enclosures whilst infected with *T. muris* also allows for the assessment of physiological and behavioural responses to resource limitation and its effect on the parasite as well as the host's immune response (Budischak *et al*., 2018).

Semi-controlled studies are opening new avenues to investigate the impact of a combination of host-intrinsic and host-extrinsic variables on the host immune response and health outcomes, and are an ideal complementation to mechanistic laboratory and observational field studies.

## The *T. muris* mouse model system – a holistic research tool

**Fig. 2.**
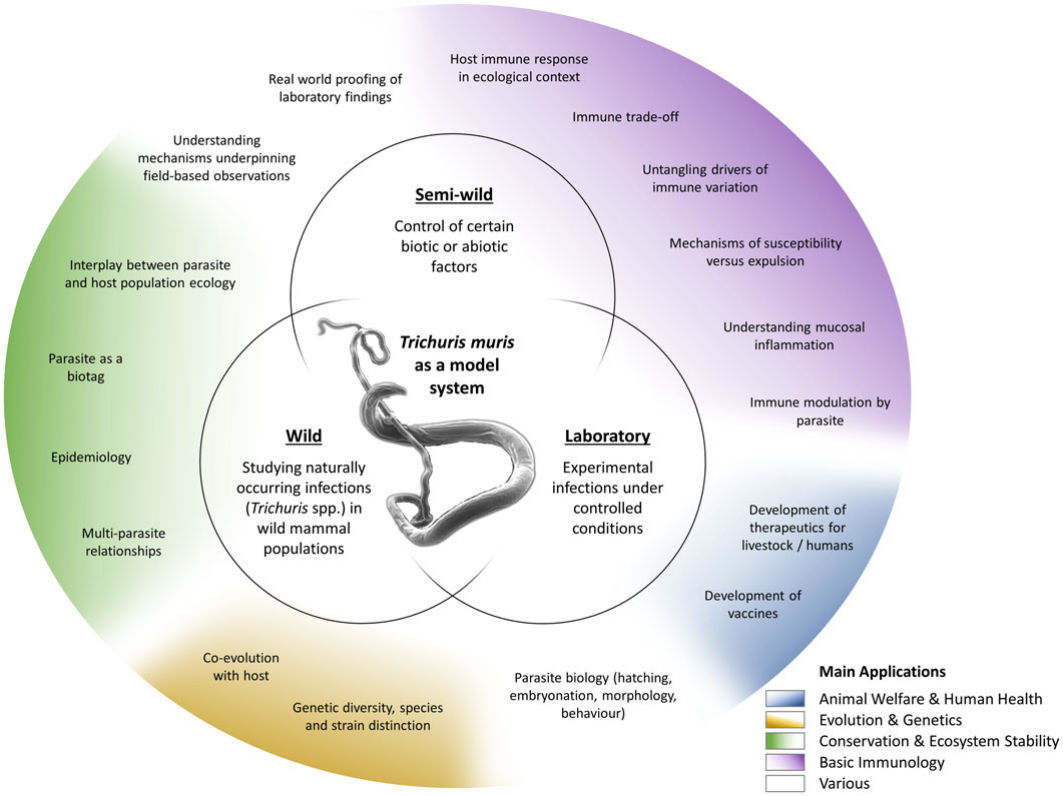
Approaches and applications of research on *T. muris*. *Trichuris* spp. and most notably *T. muris* are studied in various experimental systems (inner circles), addressing a wide variety of questions (outer circle). These fall under several broader application areas with multiple beneficiaries (colouring as explained in the legend).

The *T. muris* mouse model system offers a breadth of research opportunities across a range of disciplines (Fig. 2); a breadth arguably unique amongst the soil-transmitted helminth parasites. Through studying *T. muris* in the mouse we can gain insights into the immune responses to infection and how these immune responses underpin the ability to expel the parasite. Such studies, usually performed in laboratory settings, inform strategies for disease control as well as aiding understanding of basic immunological principles. Using *T. muris* in the mouse, we can also ask fundamental questions about parasite biology and host–parasite interactions. Naturally occurring infections with *Trichuris* spp. offer a tool for ecological and environmental research unrelated to the disease itself, for example the co-evolution of host–parasite relationships, genetic diversity and ecosystem stability, impacting our understanding of biodiversity and informing conservation efforts. Combining ecology with immunology offers new opportunities for studying whipworm (and other) infections in the house mouse in semi-wild and wild settings, which capture the complexities of context-dependent exposure with the power of working with a model organism.

These interdisciplinary approaches will open the doors for meaningful discoveries allowing us to better understand the interactions amongst the host, parasite and environment.
